# Glucagon-Like Peptide-1 Receptor Agonists for Chronic Weight Management

**DOI:** 10.1155/2023/9946924

**Published:** 2023-09-20

**Authors:** Magda Wojtara, Ashmita Mazumder, Yusra Syeda, Nikodem Mozgała

**Affiliations:** ^1^Department of Human Genetics, University of Michigan Medical School, Ann Arbor, USA; ^2^Department of Psychology, University of Toronto, Toronto, Canada; ^3^Daphne Cockwell School of Nursing, Toronto Metropolitan University, Toronto, Canada; ^4^Collegium Medicum, Jan Kochanowski University, Kielce, Poland

## Abstract

Rates of obesity have risen over the past few decades. Subsequently, the popularity of the pharmaceutical weight-loss drug market has grown over the past few years to meet growing demand. Among the most commonly prescribed drugs for weight management, many are glucagon-like peptide-1 receptor agonists (GLP-1 agonists) which are also utilized for the management of type 2 diabetes. There is a substantial and growing body of research comparing the efficacy of different clinical trials and examining long-term safety. This literature review examines the rise of off-label prescribing practices in the management of weight, with a focus on GLP-1 agonists. Physicians and patients should be aware of the unique aspects of existing treatment options, the impacts of off-label prescribing, and the effects of these medications. This review emphasizes the importance of informed decision-making, as well as the need for further research to guide future clinical practice.

## 1. Introduction and Background

Glucagon-like peptide-1 receptor agonists (GLP-1 agonists) such as semaglutide (Ozempic®) have recently risen exponentially in popularity, in part due to their prevalence on social media platforms, and celebrity endorsements. Drugs in this category have also shown promising efficacy levels in terms of average weight loss. Prescriptions for Ozempic® are up 152% compared to the last year alone [1]. Ozempic® has skyrocketed demand in the pharmaceutical space for the weight-loss drug market. Indeed, the prescription weight-loss drug market grew 72% more than initially forecasted in 2023, ballooning to a 2.3 billion dollar industry [2]. Alongside Ozempic®, the Food and Drug Administration (FDA) approved Wegovy® in June 2021, which is a higher dose formulation of semaglutide compared to Ozempic®. Wegovy® intended utilization is for adults with obesity (BMI ≥ 30 kg/m^2^) or overweight (BMI ≥ 27 kg/m^2^) who also have additional weight-related problems and adolescents with an initial BMI at or above the 95^th^ percentile for age and sex [3]. Ozempic® is intended for the treatment of type 2 diabetes but has been increasingly prescribed off-label without the benefit of an FDA-reviewed analysis of safety and efficacy data [4]. While this has allowed some to have access to the touted benefits, off-label prescribing is not without its own pitfalls. Many have voiced concerns about the availability and continuity of care interruptions inevitable for individuals with diabetes as Ozempic® continues to see growing demand. Others are troubled by the harm caused by social media platforms and celebrities heralding medications such as Ozempic® as quick, cosmetic fixes without acknowledging the side effects and the fact that discontinuation results in weight gain. Furthermore, there are very tangible side effects including hypoglycemia, gastrointestinal effects, cardiovascular risks, and drug interactions [5].

While many have popularized both Wegovy® and Ozempic®, fewer have discussed Rybelsus® and other GLP-1 agonists. Unlike Ozempic® and Wegovy®, Rybelsus® is an oral medication. As an oral medication, a 14 mg daily dose of Rybelsus® is equivalent to the 0.5 mg weekly dose of Ozempic® [6]. Rybelsus®, besides the need to be taken daily, requires that a patient consume it on an empty stomach exactly 30 minutes before eating breakfast [6]. Thus, current studies are interested in the differential efficacy, benefits, and side effects of these different forms of semaglutide. Several recent clinical studies have focused on the effectiveness of different doses and drug delivery typologies (oral, injection, etc.). Beyond this, studies have also begun comparing different GLP-1 agonists, including increasingly popular tirzepatide (a dual GLP-1/glucose-dependent insulinotropic peptide (GIP) agonist) and danuglipron.

## 2. GLP-1 Agonists for the Management of Type 2 Diabetes and Weight

### 2.1. Mechanisms of Action for GLP-1 Agonists

Glucagon-like peptide 1 (GLP-1) agonists belong to a class of type-2 diabetes medications that mimic the function of the GLP-1 hormone secreted by the pancreas [7]. GLP-1 reduces glucose levels by stimulating insulin secretion upon glucose intake, also known as the incretin effect [7]. In the same way, GLP-1 agonists bind to GLP-1 receptor sites and trigger the same effects even in the absence of intrinsic GLP-1 molecules. GLP-1 is prone to degeneration by enzyme dipeptidyl peptidase 4 (DPP-4) which reduces the overall efficacy of the hormone. However, synthetically produced GLP-1 agonists are able to evade the effect of DPP-4 (as they are administered with a DPP-4 inhibitor), thereby increasing insulin production and lowering blood glucose levels over longer periods of time [8].

### 2.2. Utilization for Type 2 Diabetes

The activity of GLP-1 deteriorates with poor glucose control in the body; hence, its efficacy is reduced further in patients with diabetes [9]. In this case, synthetically produced GLP-1 receptor agonists can substitute for intrinsically available GLP-1 receptors in patients with diabetes. Like the GLP-1 enzyme, GLP-1 receptor agonists can suppress appetite, delay gastric emptying, reduce plasma glucose levels, and enhance insulin secretion, thereby controlling glucose levels in the body [10].

### 2.3. Medical Effects and Side Effects of GLP-1 Agonists

Current research suggests that GLP-1 receptors are present throughout the body; hence, its effect extends beyond glycemic control [11]. In fact, GLP-1 receptor agonists have been linked to improved cardiac function (i.e., decreased blood pressure and lower risk of stroke or heart attack) and kidney function [11]. GLP-1 receptor agonists also contribute to glycemic control via a reduction in body weight, which is beneficial for patients with type-2 diabetes [12]. The particular effect of GLP-1 agonists on weight loss has also made it a popular drug among patients without diabetes. While there are several advantages to the medication, it is not without its side effects. Some of the more commonly experienced side effects include nausea, vomiting, and diarrhea [12]. Past research also suggests that long-term use of such medications may result in more severe side effects such as hypoglycemia (low blood sugar), pancreatitis, and C-cell hyperplasia, a potential precursor to medullary thyroid carcinoma [7, 11]. Although it is important to note the side effect of hypoglycemia impacts patients with diabetes on other antihypoglycemic agents, the overall risk for hypoglycemia in those without diabetes and not on antihypoglycemic medications is low.

### 2.4. Long-Term Efficacy of GLP-1 Agonists

In a recent observational study, researchers evaluated the efficacy of GLP-1 receptor agonists and found that on average when patients were able to continue the medication long-term (between 4 to 10 years), they maintained significantly lower HbA1c% than implementing lifestyle changes such as diet control or exercise [13]. In fact, the study also found that patients who discontinued the medication had a 30–33% increased risk of cardiac disorders compared to patients who continued to take the medication [14, 15]. In terms of weight loss, patients with diabetes on average were able to lose 4–6.2% of their total weight (and nondiabetics up to 17.4%) with long-term (>1 year) use of this medication [16]. Other studies have also reported similar results such that long-term use of GLP-1 receptor agonists was associated with consistently lower HbA1c% (up to 1.7% points lower) readings and weight loss [17].

### 2.5. GLP-1 Agonists and Lifestyle Intervention

While these medications are sometimes prescribed in isolation, some clinical studies have reported better outcomes upon combining them with lifestyle changes. For example, a recent study evaluated the effect of exercise and GLP-1 receptor agonists on weight loss, blood glucose levels, and cardiovascular outcomes [18]. When combined with exercise, patients were able to lose 3.7% of their abdominal fat and maintain normal blood glucose ratings with no increased risk for hypoglycemia and reported reduced metabolic syndrome severity, suggesting better cardiovascular health [18]. Therefore, combining this medication with exercise and/or diet may have beneficial results which have been investigated extensively in existing literature.

## 3. Semaglutide

### 3.1. FDA Approval

Semaglutide which is given subcutaneously was first approved by the FDA in 2017 for the use of type 2 diabetes management under the brand name of Ozempic® [19]. Since then, semaglutide has been approved to treat individuals with chronic obesity, to aid in weight loss. In 2019, the oral form of semaglutide, under the brand name Rybelsus®, was approved for individuals with type 2 diabetes to manage glucose control [20]. Most recently, Wegovy® was approved for weight loss in adults in June 2021 and adolescents ≥12 years old in December 2022 [21].

### 3.2. Development and Manufacturing

Currently, there are three versions of semaglutide on the market. These include Ozempic®, Wegovy®, and Rybelsus®. They differ by the amount of semaglutide sold. The company that develops and manufactures it is a global pharmaceutical company, Novo Nordisk.

### 3.3. Biochemical and Metabolic Mechanism

Semaglutide is a GLP-1 receptor agonist. This drug mimics the effect of the GLP-1 incretin hormone. The hormone binds to the GLP-1R receptor, and by doing so, it increases the sensitivity of the beta cells in the pancreas to glucose. In addition, glucagon release is also decreased by GLP-1, a hormone that increases blood sugar. GLP-1 also affects the digestive system. It slows down the movement of food in the gastrointestinal tract and regulates blood sugar after a meal [22]. It also induces the feeling of fullness after a meal, reducing appetite. Semaglutide is a 31-amino acid peptide and is mostly analogous to the GLP-1 hormone. To avoid degradation by the DPP-4 hormone, in the 8th position containing alanine, it is substituted with 2-aminoisobutyric acid (Aib) [23]. The lysine at the 34th position is acylated with a C18 fatty diacid [23]. In oral pills, salcaprozate sodium (SNAC) is added to semaglutide to increase absorption since it prevents PH breakdown [24].

### 3.4. Dosage and Drug Delivery Typologies

Semaglutide is available in oral and subcutaneous injection forms, and FDA-approved doses of semaglutide for chronic weight management are 1.7 mg or 2.4 mg weekly. It is important to note that there are other doses available (0.25 mg, 0.5 mg, 1.0 mg, and 1.7 mg) which are titration doses. Wegovy® is initially started at 0.25 mg weekly, in four-week intervals, until the 2.4 mg dose is attained [25]. Ozempic®, in the 0.5 mg and 1 mg doses and most recently in a 2.0 mg dose, is used as a once-weekly injection. In the oral form, Rybelsus® is approved in 3 mg, 7 mg, and 14 mg doses. The 3 mg dose is used once daily for 30 days before titrating up to a 7 mg dose [25].

### 3.5. Clinical Trial Results

There were several clinical trials conducted with semaglutide. Phase 3 of the semaglutide treatment effect in people with obesity or the STEP 3 program proved efficacy in reducing weight at much higher ranges at the 2.4 mg dose [26]. The study showed, along with weight loss, that the drug also improved the quality of life and well-being of participants, as cardiovascular risks such as high blood pressure were also being managed. STEP TEENS was conducted regarding the efficacy and safety of semaglutide in adolescents [26]. It also noted a few adverse effects of semaglutide, which included diarrhea and other gastrointestinal disturbances [27]. Clinical trials SUSTAIN (Semaglutide Unabated Sustainability in Treatment of Type 2 Diabetes) and PIONEER (Peptide Innovation for Early Diabetes Treatment) investigated the effect of semaglutide 1.0 mg subcutaneously as well as oral forms. SUSTAIN demonstrated that semaglutide was effective in weight loss. Furthermore, it showed it was more effective than other GLP-1 RAs such as liraglutide [28]. The PIONEER clinical trials tested the effectiveness of Rybelsus® and proved that the drug was effective against type 2 diabetes at the 7 mg and 14 mg doses in improving glycemic control and achieving a reduction in weight [28]. It is likely that fewer have discussed Rybelsus® relative to Ozempic®, given the lower average effectiveness in terms of weight loss.

## 4. Tirzepatide

### 4.1. FDA Approval

Tirzepatide, under the name Mounjaro®, was approved by the FDA on May 14, 2022, for the treatment of type 2 diabetes [29]. It is the first and only approved coagonist for GLP-1 and glucose-dependent insulinotropic peptide (GIP) [29].

### 4.2. Development and Manufacturing

Mounjaro® is developed and manufactured by Eli Lilly and Company. Its unique dual-activity property has given it the name “twincretin” [29].

### 4.3. Biochemical and Metabolic Mechanism

Tirzepatide targets GLP-1 receptors by attaching to them and activating the release of insulin. It also inhibits the secretion of glycogen and delays gastric emptying, which increases satiety [30]. Unlike other GLP-1 agonists, tirzepatide also targets glucose-dependent insulinotropic polypeptide (GIP) receptors, which help maintain homeostasis by addressing carbohydrate, lipid, and protein metabolic pathways [31]. Tirzepatide is a synthetic linear peptide molecule made up of 39 amino acids. Residues originate from GLP-1, GIP, and semaglutide, and some residues are distinct. Tirzepatide exhibits a long half-life and a strong affinity for albumin because of two noncoded amino acid residues in its peptide sequence. Given the promising results from tirzepatide, it is possible that further research in synthetic peptide therapeutics will gain momentum [29].

### 4.4. Dosage and Drug Delivery Typologies

Currently, Mounjaro® is available in the following doses: 2.5 mg, 5 mg, 7.5 mg, 10 mg, 12.5 mg, and 15 mg [32]. In pharmacokinetic studies, it was found that the mean half-life was 116.7 h (5 days); hence, the most optimal and practiced form of dosing is weekly subcutaneous injections of the drug [29].

### 4.5. Clinical Trial Results

In a phase 3 double-blind, randomized, controlled trial, it was found that after 72 weeks, patients had a mean reduction in weight of −15% with 5 mg weekly doses, −19.5% with 10 mg doses, and −20.9% with 15 mg doses, compared to −3.1% weight loss in the placebo group [33]. These participants, which largely had BMI > 30 kg/m^2^, had reductions in body weight of 10% or more, 15% or more, and 20% or more from baseline than participants in the placebo group (*P* < 0.001) [33]. Improvements in cardiometabolic measures were noted [33]. Gastrointestinal issues, which were mild to moderate in severity, were the most reported side effects and occurred primarily during dose escalation [33]. In a comparison of several studies, a mean average weight loss of −9.81 kg was reported in patients taking tirzepatide versus patients taking a placebo [32]. In all studies taken into account, hypoglycemia risk was not higher compared to a placebo [32, 33]. Apart from gastrointestinal issues, no other serious side effects have been reported [32]. These studies show that tirzepatide can be an effective treatment in chronic weight management.

## 5. Danuglipron

### 5.1. FDA Approval

Danuglipron is currently not FDA-approved as it has not passed phase three of clinical trials. Phase two clinical studies have reported the efficacy of the medication in reducing HbA1c levels and assisting weight loss, suggesting its usefulness as an antidiabetic medication [34].

### 5.2. Development and Manufacturing

Danuglipron is being developed and manufactured by Pfizer [34]. Existing GLP-1 receptor agonist therapies typically require subcutaneous administration which may be inconvenient for or not preferred by some patients, resulting in reduced adherence [35].

The production of danuglipron was motivated by the preference for oral medications over injectable alternatives by patients with diabetes [36]. Unlike other GLP-1 agonists, danuglipron is a small molecule making it a potential orally administered alternative [37].

### 5.3. Biochemical and Metabolic Mechanism

Not unlike other GLP-1 agonists, danuglipron works by mimicking hormone GLP-1 by binding to its receptor site and stimulating insulin secretion, beta cell proliferation, and slower gastric emptying [30]. On a biochemical level, according to the study by Griffith et al., danuglipron is a nonpeptide small molecule receptor agonist which contains carboxylic acid such that it gains increased affinity to bind to the GLP-1 receptor uniquely unlike other GLP-1 receptor peptide agonists [30]. Specifically, danuglipron stabilizes a previously inactive conformation of the GLP-1 receptor which involves amino acid tryptophan at position 33 of the peptide chain. Furthermore, this binding strategy is only present in primates and can only be utilized by solvents rather than peptides, making small molecules such as danuglipron the ideal candidate for use in humans [30].

### 5.4. Dosage and Drug Delivery Typologies

Currently, danuglipron is undergoing clinical trials and is being tested as an oral medication to be taken twice a day available in varying doses of 10, 20, 40, 80, or 120 mg [30]. Earlier this year, the FDA approved a label update to permit Rybelsus® first-line utilization for adult patients with type 2 diabetes. Rybelsus® can only be taken by patients 30 minutes before food. In comparison, studies have found that danuglipron can be administered before or after food, thereby making it easier for patients to adhere to this medication [34].

### 5.5. Clinical Trial Results

Danuglipron is currently undergoing phase 3 clinical trials. Results in healthy (nondiabetic) patients from phase 1 clinical trials suggest that danuglipron is tolerated well with no severe side effects. When administered at a dose of 300 mg, twice a day, patients reported side effects such as nausea and vomiting. However, no incidences of hypoglycemia, cardiac disorders, or death were reported [30]. In another phase 1 study conducted on patients with type 2 diabetes, researchers implemented an ascending dosage order starting from 10 mg twice a day to 120 mg twice a day [38]. After 28 days, researchers reported that the efficacy of danuglipron was comparable to other injectable GLP-1 agonist alternatives. While patients did not report any significant adverse effects, higher doses were associated with discomfort, nausea, and vomiting. Higher doses were also associated with an increased heart rate in patients, but other indicators, such as ECG reports, did not suggest abnormalities in these patients compared to a placebo. Most significantly, this study found that danuglipron was able to reduce HbA1c% (ranging from 0.9% to 1.2% points lower depending on dosage) and weight (2.2 kg to 7.2 kg reduced depending on dosage) after just 28 days of administration [38]. Phase 2 clinical trials on type 2 diabetic patients reported similar results. When administered over 16 weeks, danuglipron significantly reduced HbA1c% levels and resulted in weight loss in all patients [34]. This phase 2 study reported that HbA1c% levels were reduced by 1.16% points in the 120 mg (twice a day) group and they also experienced the highest weight loss (−2.04 kg) in the cohort [34]. Overall, these studies indicate that danuglipron is a promising antidiabetic drug that may increase adherence in patients given its form of delivery and ease of administration.

## 6. Other Considerations

### 6.1. Global Obesity Epidemic

The global obesity epidemic is an ongoing challenge, affecting more than 2 billion individuals [39]. The potential disease impact of obesity has been widely debated and explored in recent research. A consideration is that recent literature has shown that the correlation between the body mass index and disease risk is not linear [39]. In fact, BMI only accounts for ∼17% of the risk of insulin resistance and subsequent type 2 diabetes in BMI ≥ 25 kg/m^2^ population [39]. It is possible that other factors such as socioeconomic status, access to preventative care, and epigenetics may play a larger role in disease risk. However, future studies should address these links as they pertain to specific diseases and illnesses in clearly defined contexts.

As global obesity rates continue to rise, many individuals are exploring solutions beyond lifestyle changes to address weight management. The prevailing approach for weight management is through a combination of diet, exercise, and pharmacotherapy [40]. In some cases, metabolic/bariatric surgery may be considered an appropriate treatment for long-term weight loss. However, it is difficult to draw overarching recommendations given the heterogeneity of underlying causes of obesity and weight gain. It is important that research is ongoing to determine the most appropriate protocols for the management of weight in different populations, including individuals with different hormone imbalance-induced weight gain including ghrelin, leptin, amylin, PYY, and thyroid hormones.

### 6.2. The Impact of Social Media

High social media usage has been commonly associated with a negative self-perception of body image and the development of illnesses such as depression and eating disorders [41]. Some have also argued that social media influencers and technology such as “filters” have contributed to an uptick in some elective cosmetic procedures. For instance, a study found that more than half of respondents who underwent rhinoplasty did so due to social media “before and after” advertisements [42]. Despite this, social media is a great potential avenue for the propagation of public health-informed recommendations to mitigate obesity [43]. Social media platforms can also be a source of social support, which is often critical to improved health outcomes for a variety of diseases. This may contribute to the significant weight loss as a result of social media-delivered weight loss interventions compared to traditional methods such as brochures and questionnaires [43].

### 6.3. Off-Label Prescribing Practices

GLP-1 agonists are typically approved for the management of conditions such as type 2 diabetes and in some cases weight management but are growing in popularity for their off-label utilization. Obesity is largely considered a risk factor for the development of type 2 diabetes. Off-label prescriptions account for 10–20% of all prescriptions written, so this practice is not uncommon [44]. Off-label prescribing, when considering a patient's individual needs, may be beneficial. Some off-label prescribing even has accumulated a growing body of high-quality evidence. Specifically, Ozempic® at lower doses of 0.25, 0.5, and 1.0 mg is like Wegovy® except for the label and device. Given ongoing supply chain issues with Wegovy®, which have primarily impacted lower doses, it may make sense to utilize Ozempic® off-label to address these shortages. There is also growing evidence for off-label antiobesity medications such as metformin and topiramate [45].

There are concerns that have been voiced by critics of off-label GLP-1 agonist-prescribing practices. One concern is that off-label prescribing has skyrocketed. Some concerns include that there has been limited research finalized on the effective, safe use of these medications in children [46]. This may be attributed to the relatively recent approval of several of these medications in adults and current ongoing trials such as STEP YOUNG for Wegovy® and SCALE KIDS for Saxenda® [47]. Another concern is that the FDA has banned direct-to-consumer marketing of off-label uses in patient education materials and advertisements, but off-label marketing by pharmaceutical companies is a common cause of Medicaid fraudulent claim investigations [44]. Others have called to question the utilization of these drugs off-label for individuals that are not in the categories of a BMI above 30 or above a BMI of 27 with another weight-related condition threatening their health. While many studies have demonstrated efficacy in patients with diabetes and obesity, a worrying trend is interest in Ozempic for cosmetic weight loss popularized by social media and celebrity influences [48]. Furthermore, off-label medication could interact with other medications and worsen other health conditions and may not have rigorous data to support its safety and effectiveness short or long term in different patient populations.

## 7. Conclusion

GLP-1 agonists such as semaglutide (Ozempic® and Wegovy®) have recently risen exponentially in popularity. This rise in popularity can be attributed to successes demonstrated by clinical trials and the prevalence of discussions regarding this on social media. To our knowledge, previous studies have not explored similarities and differences among other GLP-1 agonists, different drug delivery typologies (oral and injection), and dosages focusing on their off-label utilization and results of the most recent clinical trials. Healthcare providers must make sure to consider the unique history, needs, and goals of their patients before prescribing a GLP-1 agonist. There are many diagnostic tools and management options that may be used in conjunction with GLP-1 agonists, and the efficacy of different combinations of drugs and lifestyle changes should also be further explored. It is crucial to also consider that many individuals, including some celebrities, are interested in utilizing GLP-1 agonists for aesthetic or cosmetic weight loss rather than for a truly medically needed purpose. Future research should explore the differential impacts of GLP-1 agonists in different populations, in individuals with different lifestyle habits, and long-term efficacy data to develop precision medicine recommendations.

## Data Availability

The data used to support the findings of this study are cited within the study and available from the corresponding author upon reasonable request.
